# Supplementary material for: In Silico Discovery of Potential Inhibitors Targeting the RNA Binding Loop of ADAR2 and 5-HT2CR from Traditional Chinese Natural Compounds
Source: Int J Mol Sci. 2023 Aug 9;24(16):12612. doi: 10.3390/ijms241612612 (PMC10454645; doi:10.3390/ijms241612612)
Supplement: Supplementary file 1 [file ijms-24-12612-s001.zip › ijms-2510503-supplementary.pdf]

Supplementary Information

In Silico Discovery of Potential Inhibitors Targeting the RNA Binding Loop of ADAR2 and 5-HT2CR from Traditional Chinese Natural Compounds

Emmanuel Broni [1], Carolyn Ashley [1], Miriam Velazquez [1,2], Sufia Khan [1,3], Andrew Striegel [1,4], Patrick O. Sakyi [4,5], Saqib Peracha [1], Kristeen Bebla [1,2], Monsheel Sodhi [2], Samuel K. Kwofie [7,8], Adesanya Ademokunwa [1,9], Whelton A. Miller III [1,5*]

[1] Department of Medicine, Loyola University Medical Center, Loyola University Chicago, Maywood, IL, 60153, USA; ebroni@luc.edu (E.B.); cashley1@luc.edu (C.A.); skhan96@luc.edu (S.K.); astrieg2@nd.edu (A.S.); speracha@luc.edu (S.P.); mvelazquez4@luc.edu (M.V.); kbebla@luc.edu (K.B.); aademokunwa@luc.edu (A.A.)
[2] Department of Molecular Pharmacology & Neuroscience, Loyola University Medical Center, Loyola University Chicago, Maywood, IL, 60153, USA; msodhi@luc.edu (M.S.)
[3] Department of Biology, Loyola University Chicago, Chicago, IL 60660, USA;
[4] Department of Chemical and Biochemistry, College of Science, University of Notre Dame, Notre Dame, IN 46556 USA;
[5] Department of Chemistry, School of Physical and Mathematical Sciences, College of Basic and Applied Sciences, University of Ghana, P. O. BOX LG 56, Legon, Accra, Ghana;
[6] Department of Chemical Sciences, School of Sciences, University of Energy and Natural Resources, Box 214, Sunyani, Ghana; patrick.sakyi@uenr.edu.gh (P.O.S.)
[7] Department of Biomedical Engineering, School of Engineering Sciences, College of Basic & Applied Sciences, University of Ghana, PMB LG 77, Legon, Accra, Ghana; skkwofie@ug.edu.gh (S.K.K.)
[8] Department of Biochemistry, Cell and Molecular Biology, West African Centre for Cell Biology of Infectious Pathogens, College of Basic and Applied Sciences, University of Ghana, P.O. Box LG 54, Accra, Ghana;
[9] Department of Cognitive and Behavioral Neuroscience, Loyola University Chicago, Chicago, IL 60660, USA;
* Correspondence: wmiller6@luc.edu (W.A.M., III)

Content

Table S1: Toxicity risk prediction for the top 121 compounds and known inhibitor, 8-Azanebularine.

Figure S1: 2D representation of the protein-ligand interaction maps for ADAR2 complexed with (a) ZINC000095913861, (b) ZINC000085996580, (c) ZINC000070454467, (d) ZINC000042890265, and (e) ZINC000039183320.
Figure S2: 3D representation of the protein-ligand interaction for ADAR2 complexed with (a) ZINC000095913861, (b) ZINC000085996580, (c) ZINC000070454467, (d) ZINC000042890265, and (e) ZINC000039183320.
Figure S3: Root mean square fluctuation (RMSF) plot of the ADAR2-ligand complexes throughout the 100 ns MD simulations.
Figure S4: Per-residue energy decomposition of the ADAR2 in complex with (a) ZINC000042890265, (b) ZINC000039183320, (c) ZINC000101100339, (d) ZINC000085593577, (e) ZINC000085532375, and (f) 8-Azanebularine.

Table S1: Toxicity risk prediction for the top 121 compounds and known inhibitor, 8-Azanebularine.

**Compound ID**	**Toxicity Risk**			
	**Mutagenic**	**Tumorigenic**	**Reproductive Effective**	**Irritant**
8-Azanebularine	None	None	None	None
ZINC000004098302	None	None	High	None
ZINC000004098448	None	None	High	None
ZINC000005158749	None	None	None	None
ZINC000013310993	High	None	Low	None
ZINC000014637370	None	None	High	None
ZINC000014686335	High	None	High	None
ZINC000014825188	None	None	High	None
ZINC000015253910	None	None	None	None
ZINC000033832412	None	None	None	None
ZINC000039183320	None	None	None	None
ZINC000059586224	None	None	High	None
ZINC000059587555	None	None	High	None
ZINC000070451127	None	None	None	None
ZINC000070454885	None	None	None	None
ZINC000085532375	None	None	Low	None
ZINC000085543123	None	None	None	High
ZINC000085547677	High	Low	High	High
ZINC000085547700	Low	Low	High	High
ZINC000085569457	None	None	High	High
ZINC000085592995	None	None	None	None
ZINC000085593577	None	None	High	None
ZINC000095912378	None	None	None	High
ZINC000095912380	None	None	None	High
ZINC000095912466	None	None	None	None
ZINC000095912676	None	None	High	None
ZINC000095914813	None	None	High	None
ZINC000101100339	None	None	None	None
ZINC000103559699	High	High	None	High
ZINC000103572309	None	None	None	None
ZINC000103584057	None	None	None	High
ZINC000013384051	None	None	High	None
ZINC000015206058	None	None	High	None
ZINC000033830107	None	None	High	None
ZINC000042802834	Low	None	None	None
ZINC000042890265	None	None	High	None
ZINC000044031885	None	None	None	None
ZINC000044086846	Low	None	None	None
ZINC000044305204	None	None	None	None
ZINC000044305748	None	None	None	None
ZINC000044352188	None	None	None	None
ZINC000059589111	None	None	None	None
ZINC000070450948	None	None	None	None

ZINC000070451048	None	None	None	None
ZINC000070454051	None	None	High	High
ZINC000070454074	None	None	None	None
ZINC000070454124	None	None	None	None
ZINC000070454467	None	None	None	None
ZINC000070454501	None	None	None	None
ZINC000085504890	None	None	None	None
ZINC000085509705	None	None	High	None
ZINC000085530461	None	None	None	High
ZINC000085530478	None	None	None	High
ZINC000085530480	None	None	None	High
ZINC000085530487	None	None	None	None
ZINC000085530490	None	None	None	None
ZINC000085530497	None	None	None	High
ZINC000085530502	None	None	None	None
ZINC000085531494	None	None	None	None
ZINC000085531496	None	None	None	None
ZINC000085532213	None	None	None	None
ZINC000085532258	None	None	None	None
ZINC000085532309	None	None	None	None
ZINC000085532312	None	None	None	None
ZINC000085532324	None	None	None	None
ZINC000085532381	None	None	None	None
ZINC000085532390	None	None	None	None
ZINC000085532442	None	None	None	None
ZINC000085532515	None	None	None	None
ZINC000085532517	None	None	None	None
ZINC000085532519	None	None	None	None
ZINC000085541078	None	None	None	High
ZINC000085543212	None	None	None	None
ZINC000085543539	None	None	None	None
ZINC000085543544	None	None	None	None
ZINC000085543571	None	None	None	None
ZINC000085548190	High	Low	High	Low
ZINC000085567825	High	None	High	None
ZINC000085592927	None	None	None	None
ZINC000085592968	None	None	None	None
ZINC000085592982	None	None	None	None
ZINC000085593001	None	None	None	None
ZINC000085594038	None	Low	High	High
ZINC000085594040	None	Low	High	High
ZINC000085594044	None	Low	High	High
ZINC000085594048	None	None	High	High
ZINC000085594057	None	Low	High	High
ZINC000085594065	Low	Low	High	High
ZINC000085597267	None	None	None	None

ZINC000085597294	None	None	None	None
ZINC000085597296	None	None	None	None
ZINC000085628518	None	None	None	None
ZINC000085631917	None	None	None	None
ZINC000085633079	None	None	High	None
ZINC000085761575	High	None	None	High
ZINC000085854118	None	None	None	None
ZINC000085932869	None	None	High	None
ZINC000085976998	High	High	None	High
ZINC000085991498	Low	None	None	None
ZINC000085996580	None	None	High	None
ZINC000095910037	None	None	High	None
ZINC000095910145	Low	None	None	None
ZINC000095910492	Low	None	None	High
ZINC000095910794	None	None	None	None
ZINC000095911347	None	None	None	None
ZINC000095912705	None	None	None	High
ZINC000095913861	None	None	High	None
ZINC000095914212	Low	Low	None	None
ZINC000095914586	None	None	None	None
ZINC000095914856	High	None	None	None
ZINC000095915041	None	None	None	None
ZINC000095918631	Low	None	None	None
ZINC000095919317	None	None	None	None
ZINC000103526876	None	None	None	High
ZINC000103527856	Low	None	High	High
ZINC000103527863	None	Low	High	High
ZINC000103527948	Low	None	High	High
ZINC000103543220	None	Low	None	Low
ZINC000103568616	Low	None	None	None
ZINC000103572332	None	None	None	High
ZINC000103578914	None	High	None	Low
ZINC000103585067	None	None	None	None

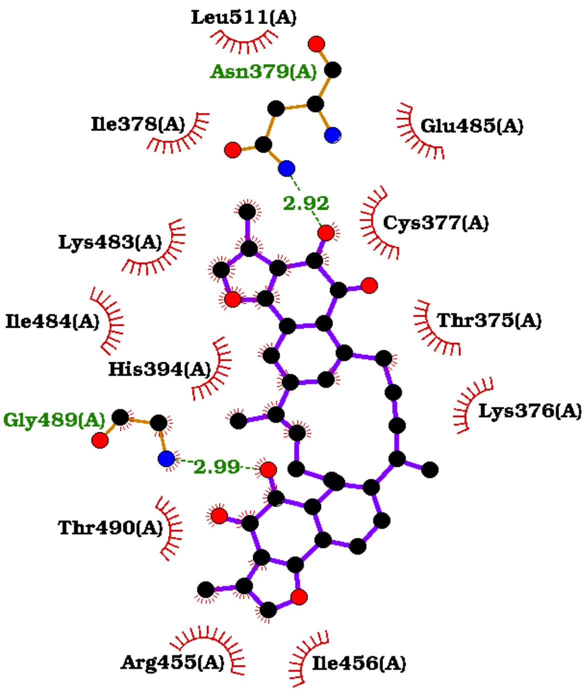
Leu511(A)
Asn379(A)
Ile378(A)
Glu485(A)
2.92
Lys483(A)
Cys377(A)
Ile484(A)
Thr375(A)
His394(A)
Lys376(A)
Gly489(A)
2.99
Thr490(A)
Arg455(A)
Ile456(A)

(a)

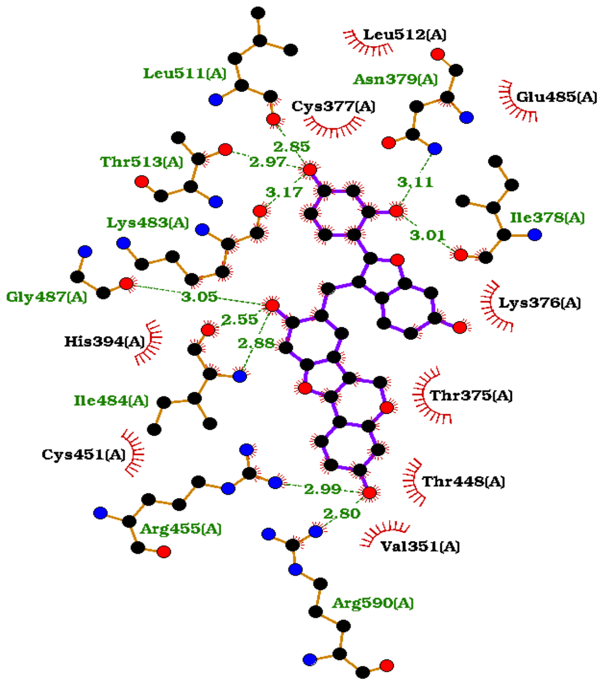
Leu512(A)
Leu511(A)
Asn379(A)
Glu485(A)
Cys377(A)
2.85
Thr513(A)
2.97
3.11
3.17
Lys483(A)
Ile378(A)
3.01
Gly487(A)
3.05
Lys376(A)
2.55
His394(A)
2.88
Ile484(A)
Thr375(A)
Cys451(A)
Thr448(A)
2.99
2.80
Arg455(A)
Val351(A)
Arg590(A)

(b)

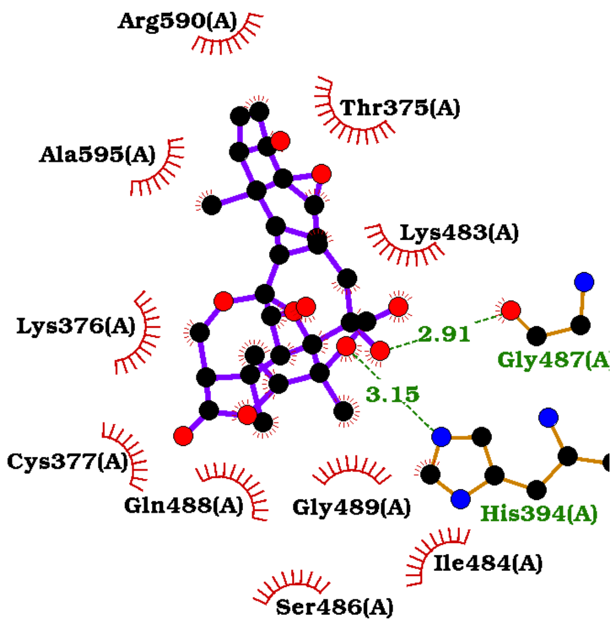
Arg590(A)
Thr375(A)
Ala595(A)
Lys483(A)
Lys376(A)
2.91
Gly487(A
3.15
Cys377(A)
Gln488(A)
Gly489(A)
His394(A)
Ile484(A)
Ser486(A)

(c)

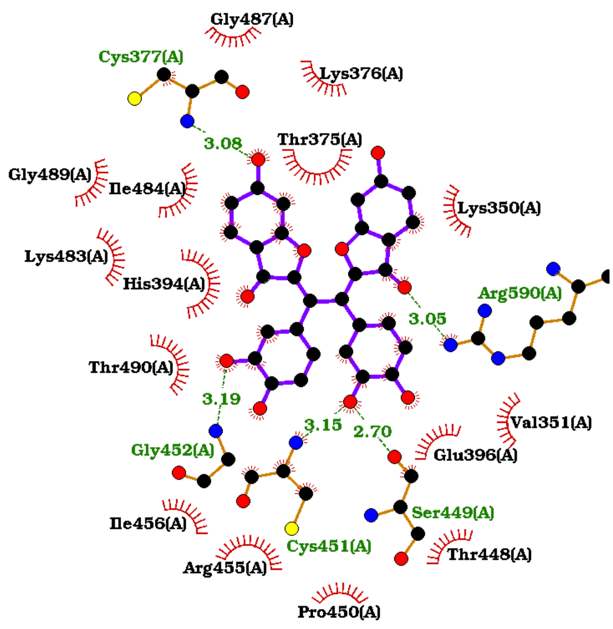
Gly487(A)
Cys377(A)
Lys376(A)
3.08
Thr375(A)
Gly489(A)
Ile484(A)
Lys350(A)
Lys483(A)
His394(A)
Arg590(A)
3.05
Thr490(A)
3.19
3.15
2.70
Val351(A)
Gly452(A)
Glu396(A)
Ser449(A)
Ile456(A)
Cys451(A)
Thr448(A)
Arg455(A)
Pro450(A)

(d)

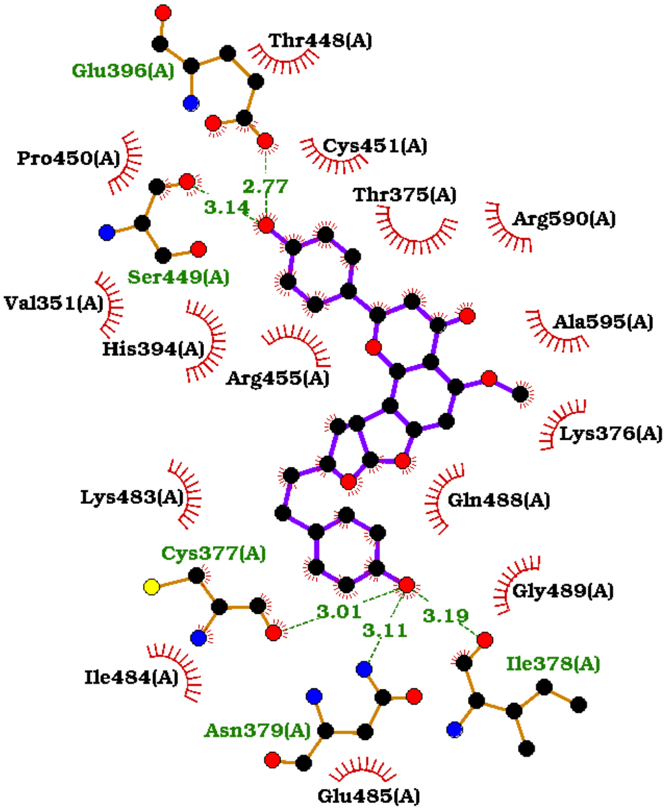

(**e**)

Figure S1: 2D representation of the protein-ligand interaction map for ADAR2 complexed with (a) ZINC000095913861, (b) ZINC000085996580, (c) ZINC000070454467, (d) ZINC000042890265, and (e) ZINC000039183320.

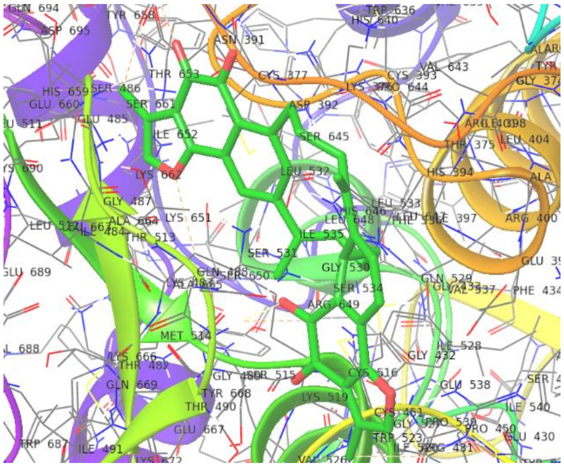

(**a**)

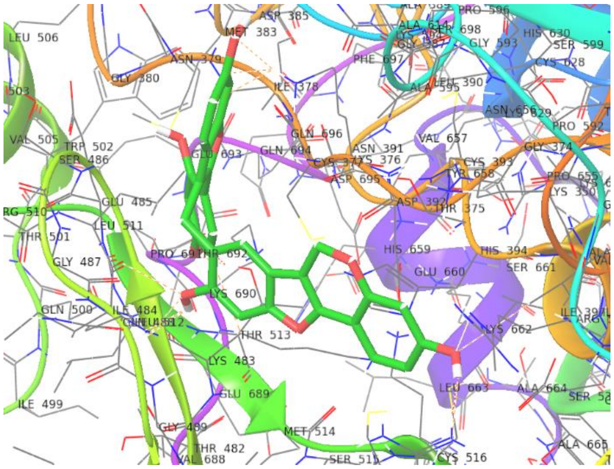

(**b**)

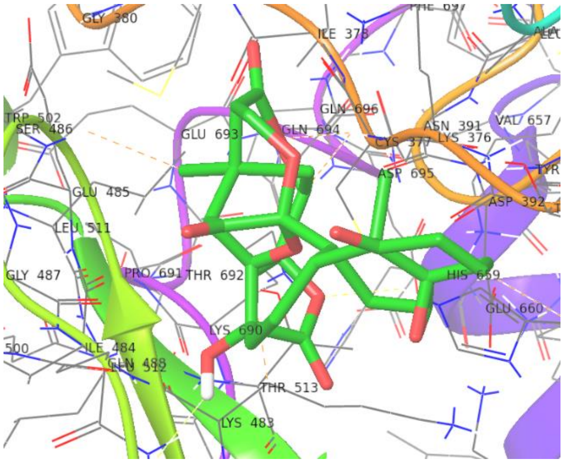

(**c**)

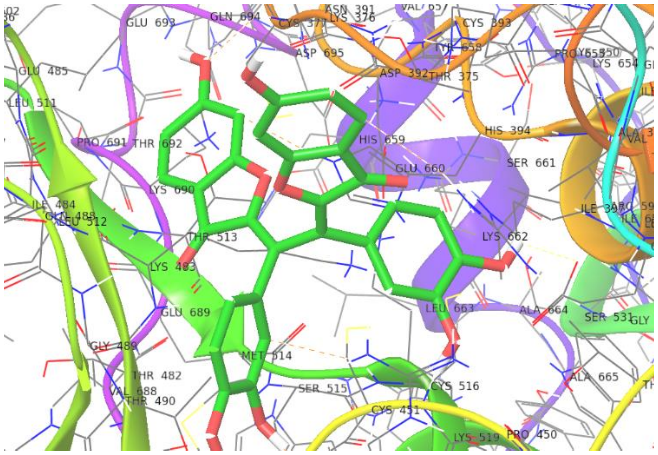

(**d**)

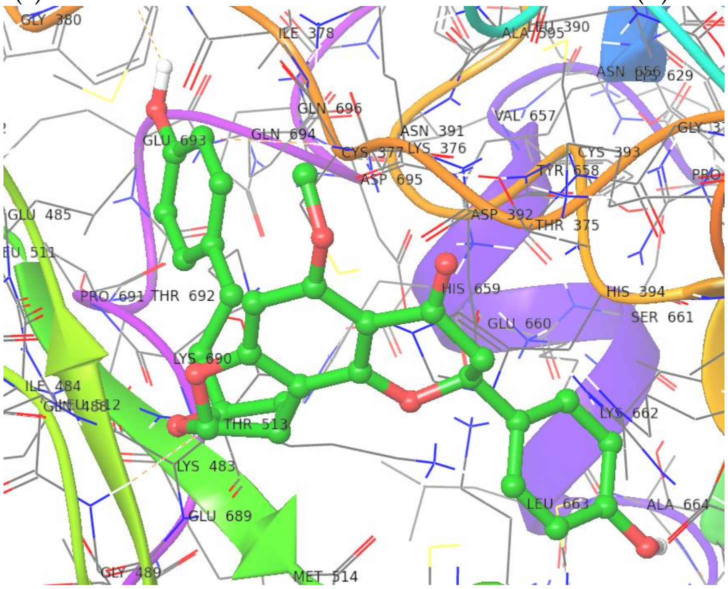

(**e**)

Figure S2: 3D representation of the protein-ligand interaction for ADAR2 complexed with (a) ZINC000095913861, (b) ZINC000085996580, (c) ZINC000070454467, (d) ZINC000042890265, and (e) ZINC000039183320.

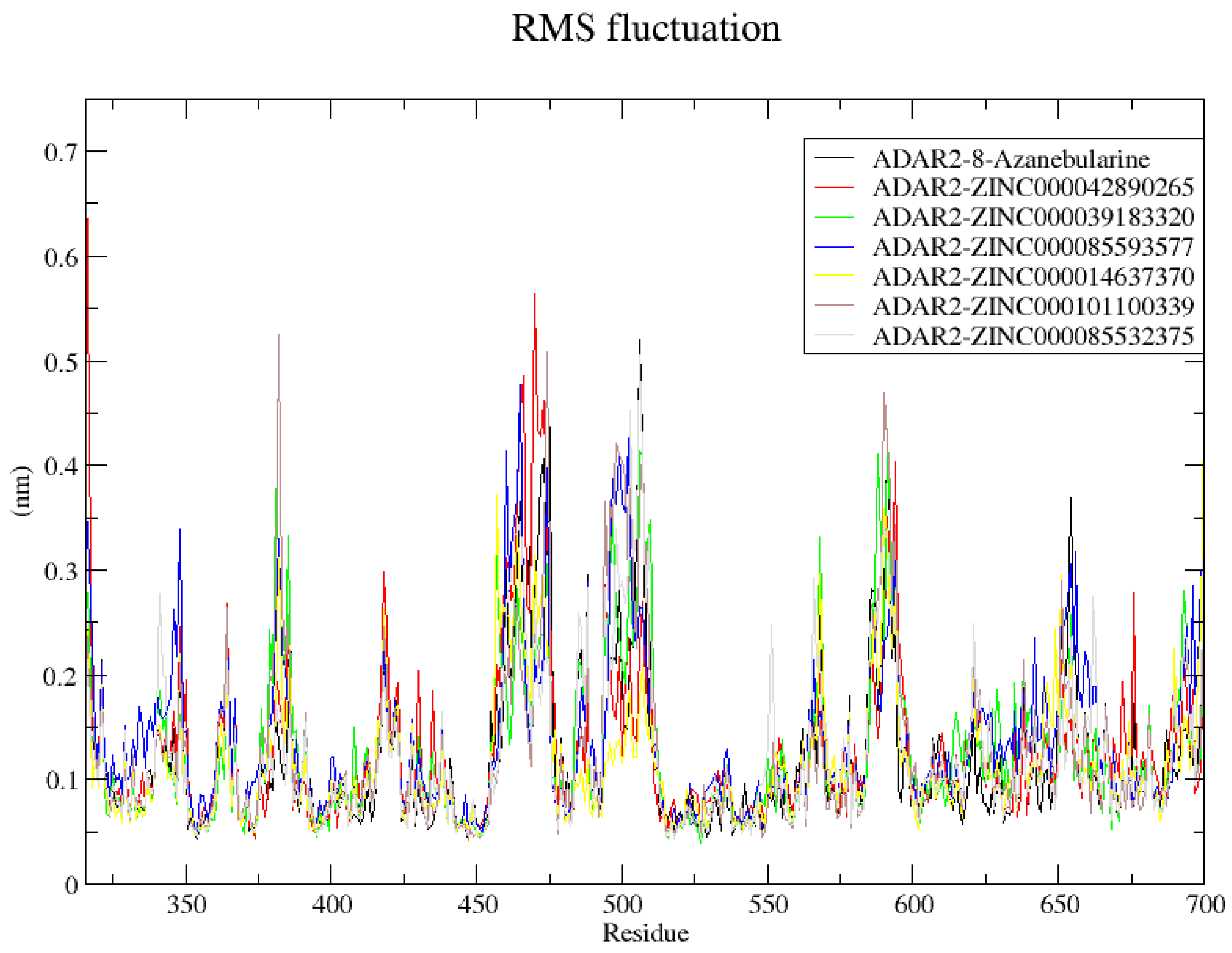

Figure S3: Root mean square fluctuation (RMSF) plot of the ADAR2-ligand complexes throughout the 100 ns MD simulations.

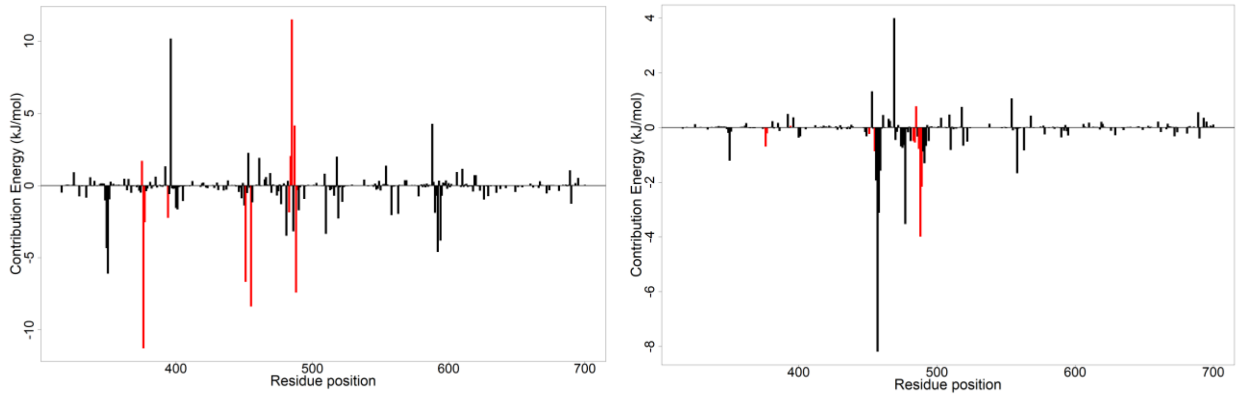

(**a**) (**b**)

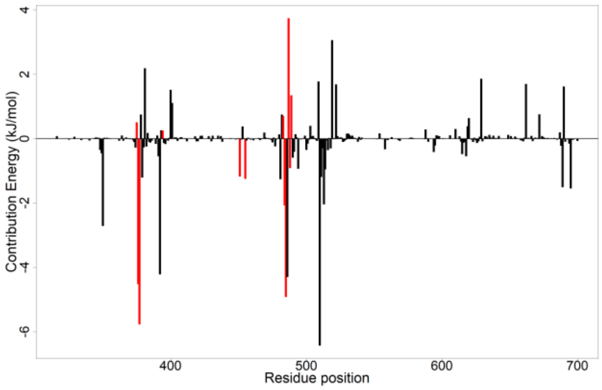

(**c**)

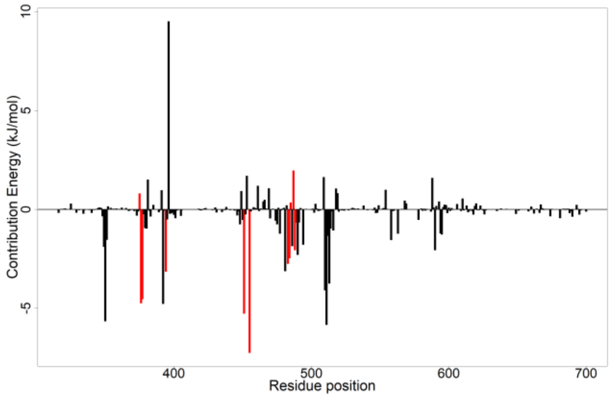

(**d**)

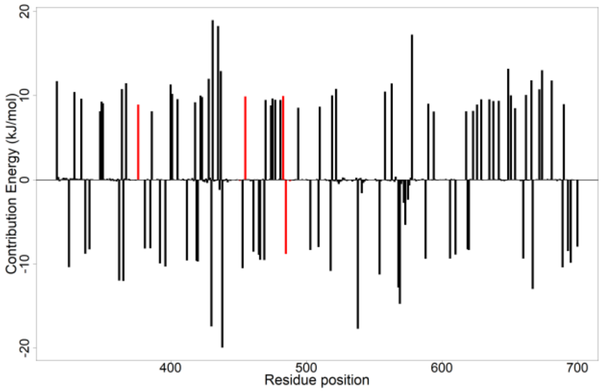

(**e**)

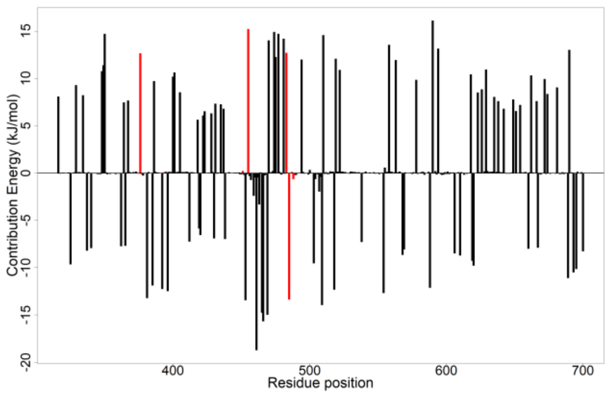

(**f**)

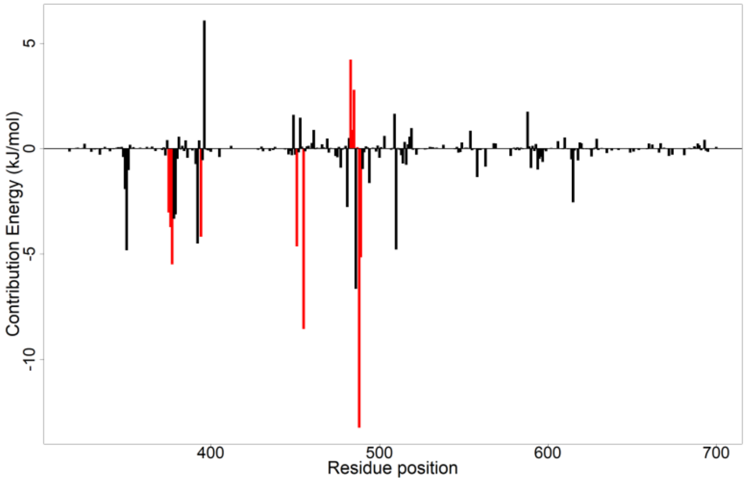

(**g**)

Figure S4: Per-residue energy decomposition of the ADAR2 in complex with (a) ZINC000042890265, (b) ZINC000039183320, (c) ZINC000101100339, (d) ZINC000085593577, (e) ZINC000085532375, (f) 8-Azanebularine, and (g) ZINC000014637370.
